# Measles outbreak amidst COVID-19 pandemic in Africa: grappling with looming crises

**DOI:** 10.1186/s41182-021-00375-3

**Published:** 2021-11-02

**Authors:** Abdullahi Tunde Aborode, Abdulhammed Opeyemi Babatunde, Bright-Agbonze Samuel Osayomwanbor, Emmanuel Adebowale Fajemisin, Oko Christian Inya, Olayinka Olajiga, Anthony Chinonso Uwandu-Uzoma

**Affiliations:** 1 Healthy Africans Platform, Research and Development, Ibadan, Nigeria; 2 Student Against COVID-19, Research and Education, New York, USA; 3 Department of Medicine and Surgery, Faculty of Clinical Sciences, College of Medicine, University of Ibadan, Ibadan, Nigeria; 4 Infection Prevention and Control Programme, School of Nursing and Health Science, University of Dundee, Dundee, Scotland UK; 5 Department of Biochemistry, Federal University of Technology, Akure, Nigeria; 6 Institute of Applied Health Research, University of Birmingham, Birmingham, UK; 7 Department of Entomology, Kansas State University, Manhattan, USA; 8 Faculty of Health studies, School of Nursing and Healthcare Leadership, University of Bradford, West Yorkshire, UK

**Keywords:** COVID-19, Measles, Africa countries, Vaccination, Double burden, Pandemic

## Abstract

Coronavirus disease 2019 (COVID-19) and measles are major threats to the health and wellbeing of Africans. Measles is an endemic disease in Africa with a high mortality rate especially in children despite available vaccines. This letter aims to discuss the impact of the COVID-19 pandemic on prevention and management of measles in Africa. The emergence of COVID-19 has exacerbated the morbidities of measles due to multi-factors like the disruption of mass measles routine vaccination, a monopolistic focus on COVID-19 eradication, malnutrition, and poor surveillance. Currently, the COVID-19 pandemic and looming measles epidemic pose a double burden on the African health sector. We recommend urgent interventions from government and other stakeholders including community leaders to strengthen measles research and vaccination programs in Africa amidst the pandemic.

## Introduction

Coronavirus disease 2019 (COVID-19) and measles are a huge burden on the health systems of Africa. Measles is caused by the Rubeola virus causing immunosuppression (i.e., fever, seizures, cough, flu, blindness, rash, diarrhea, pneumonia, and brain inflammation) that lead to fatality mainly among children below 14 years. It is the most transmittable virus ever known. The estimated mortality due to measles according to reports from the weekly epidemiology reports has increased from 89,780 in 2016 to 207,500 in 2019 [1]. Likewise, COVID-19 is caused by novel coronavirus inflicting respiratory illness outbreak with 1.81 million deaths in the 1st year [2]. Since its emergence in China, it has been declared a global health threat.

Despite the mortality rate from several measles outbreaks, measles remains one of the most vaccine-preventable diseases, which has also yielded tremendous results over the years in Africa—an improvement in vaccination coverage has led to decreased incidence from 145 to 120 cases per million populations between 2000 and 2019 [1, 3]. Although the target set by the World Health Assembly to eradicate measles by 2020 was not achieved [1, 3]. This is partly due to non-immunization or partial immunization of children in the poor/underserved communities, malnutrition, vaccine hesitancy, poor surveillance, and weak health care systems in Africa. Currently, the emergence of the Coronavirus disease in 2019 has escalated these risk factors which have generated a double burden and looming resurgence of measles epidemics which can lead to an African pandemic if not addressed.

The World Health Organization (WHO) had recommended a 95% vaccination for herd immunity against measles. However, according to a retrospective multi-country series analysis of national immunization coverage and case surveillance data in 15 West African countries carried out by Wariri et al., the measles-containing vaccine first dose (MCV1) coverage range from 45% in 2001 to 66% in 2019 and only 7 out of 15 countries has introduced a second dose of measles-containing vaccine (MCV2), since 2015 [3].

Furthermore, according to a proposed scorecard to measure the performance of countries regarding measles control and elimination milestones, it is worrisome that only three (Cape Verde, The Gambia, and Ghana) of 15 countries can be considered to have made substantial progress towards measles elimination. This disposition has gross effects in Central Africa too; for example, in Cameroon, the vaccine coverage was 71·1% for MCV1 and 13·2% for MCV2 in June 2020 [4]. Of the 190 health districts in Cameroon, 79 were affected by measles epidemics in 2020, with more than 1462 confirmed cases. Moreover, 74% of the confirmed cases were not vaccinated and supplementary immunizations activities (SIAs) were only effective in 44 (56%) of 79 health districts, very far from the elimination milestones [3, 5].

The emergence of the COVID-19 pandemic has severely strained the health system in Africa in several ways including diminishing healthcare performance and resources. The COVID-19 restriction protocols and lockdown measure has disrupted childhood immunization. According to the World Health Organization, an estimated 80 million children in 68 countries are at risk of developing vaccine-preventable diseases such as measles, diphtheria, and polio, because of the disruption of routine immunization services. This is not surprising, as the immunization system relies on functioning health facilities and stable communities to be effective. The WHO recommended the suspension of mass vaccination campaigns to prevent the worsening of community transmission of COVID-19 and this could have an immediate effect on immunization coverage, especially in rural and underserved communities [6]. Lastly, the COVID-19 pandemic has also worsened the implementation of mass measles vaccination, hence increasing the population of unimmunized children susceptible to measles resulting in a double burden and gross fatality rate.

## Recommendations

To address these issues, it is important that the African Union (AU) and African Centre of Disease Control (CDC) provide leadership and management training to the teams to adequately use strategic problem solving, political advocacy, and other tools to find innovative and homegrown solutions to prevent measles amidst the COVID-19 era. Furthermore, there is a need for African countries at the national health level to improve their disease surveillance and report yearly progress to WHO and Africa Centre for Disease Control and Prevention for a concerted effort [3, 5].

In the localized (rural) region where partial or non-immunization of children is rampant, there is a need to reach unimmunized children through catch-up vaccination schedules and campaigns, including supplementary immunization activities [1]. During this engagement, community health workers should adhere strictly to COVID-19 prevention protocols [5].

Therefore, there is need for the government and every health stakeholders to address vaccine hesitancy—via implementing an inclusive community engagement strategy that will involve accurate knowledge on vaccination, debunking religious and cultural myths towards vaccines and integrating a vaccination framework with the help of cultural, religious and community leaders. Adopting a native framework where community health workers can integrate local languages to disseminate information and risk communication can improve community knowledge on vaccines and improve confidence in vaccination [7, 8].

The COVID-19 pandemic has exposed the vulnerability of the African health sector, and efforts that are more concerted should be to restructure the health sector to prepare for other expected outbreaks. Every stakeholder in the African health sector must not lose sight of measles and rubella elimination targets and implement the new Measles and Rubella Strategic Framework 2021–2030 [5]. One important key to meeting this target is adequate funding. As much as the COVID-19 pandemic is a burden, the joint World Health Organization–United Nations Children Funds (WHO–UNICEF) and relevant stakeholders must prioritize other infectious diseases like measles ravaging children financially.

## Conclusion

The double burden caused by the COVID-19 pandemic and measles is due to the disruption in immunization campaigns and vaccination coverage across Africa because of the COVID-19 restriction protocols and lockdowns. Tackling the measles outbreak amidst COVID‐19 entails revamping and strengthening the Africa’s health systems with a focus on essential medical services and life-saving vaccination facilities to create robust immunization programs that can efficiently and effectively run, regardless of any disease outbreak.

## Data Availability

Not applicable.

## Abbreviations

COVID-19: Coronavirus 2019
WHO–UNICEF: World Health Organization–United Nations Children Funds
WHO: World Health Organization
MCV1: Measles-containing vaccine first dose
MCV2: Measles-containing vaccine second dose
AU: African Union
CDC: Center of Disease Control
